# Development and pilot implementation of CERMATA: A digital referral-based vision screening platform for Indonesian primary school children

**DOI:** 10.1371/journal.pone.0359572

**Published:** 2026-09-30

**Authors:** Kianti Raisa Darusman, Alicia Kartika, Christella Caroline, Tanessa Audrey Wihardji, Bunga Pelangi, Ray Wagiu Basrowi, Nila Faried Moeloek

**Affiliations:** 1 Indonesia Health Development Center (IHDC), Jakarta, Indonesia; 2 Health Collaborative Center (HCC), Jakarta, Indonesia; 3 Indonesian Ophthalmologist Association (PERDAMI), Jakarta, Indonesia; 4 Faculty of Medicine, Universitas Indonesia, Jakarta, Indonesia; University of Warmia, POLAND

## Abstract

**Background:**

Refractive error is a leading treatable cause of visual impairment among school-aged children, yet implementation of standardized school-based vision screening remains inconsistent in many low- and middle-income countries. CERMATA is a web-based digital platform that integrates the validated WHOeyes mobile application with referral management and program monitoring.

**Methods:**

This cross-sectional pilot implementation study evaluated the operational feasibility and preliminary screening performance of CERMATA in two primary schools participating in the initial implementation phase of the CERMATA program in Jakarta, Indonesia. Seventy-two children aged 9–12 years completed both CERMATA screening and standardized Snellen visual acuity assessment on the same day. Referral-level reduced visual acuity was defined as presenting visual acuity worse than 6/9 in either eye. Screening performance, agreement, and receiver operating characteristic (ROC) analysis were evaluated at the participant level using the poorer eye.

**Results:**

According to the Snellen reference assessment, 58 participants (80.6%) met the referral criterion. CERMATA demonstrated a sensitivity of 96.6%, specificity of 71.4%, positive predictive value of 93.3%, negative predictive value of 83.3%, and overall accuracy of 91.7%. Agreement with the reference assessment was substantial (κ = 0.719), and ROC analysis demonstrated excellent discrimination (AUC = 0.914; 95% CI, 0.83–1.00). All participants included in the screening performance analysis completed both assessments, supporting the operational feasibility of the screening workflow.

**Conclusion:**

In this pilot implementation study, CERMATA demonstrated promising screening performance and operational feasibility for identifying reduced vision in older primary school-aged children. Larger multicenter implementation studies are warranted to confirm its performance, scalability, and integration into routine eye-care pathways before national-scale implementation.

## Introduction

Reduced visual acuity (VA) among school-aged children remains an important public health concern worldwide because of its impact on educational attainment, psychosocial well-being, and long-term productivity [1,2]. Reduced VA may arise from a variety of ocular conditions, including uncorrected refractive error (URE), amblyopia, media opacity, and retinal or neurological disorders [3]. Among these, URE is consistently reported as the most common and readily treatable cause of reduced VA among school-aged children and is responsible for a substantial proportion of avoidable visual impairment worldwide [3,4]. An estimated 12.8 million children aged 5–15 years are affected by visual impairment attributable to URE [5]. Childhood reduced VA, particularly when caused by URE, has been associated with poorer academic performance, reduced classroom participation, impaired psychosocial well-being, and diminished vision-related quality of life [6]. Because visual deficits often develop gradually and may not be recognized by children, parents, or teachers, many affected children remain unidentified until visual difficulties begin to interfere with learning and everyday activities [7]. This delayed recognition is particularly concerning during the school years, when increasing educational demands make adequate visual function essential for academic participation and achievement.

Despite the availability of effective interventions, many children with reduced VA remain undetected, particularly in low- and middle-income countries where access to eye care services remains limited [8]. In Indonesia, school-based vision screening is incorporated into the national School Health Program (*Usaha Kesehatan Sekolah*, UKS); however, implementation remains inconsistent because of limited human resources, competing health priorities, inadequate screening equipment, and restricted access to trained eye care personnel [9,10]. As a result, screening activities frequently depend on teachers or primary healthcare workers with limited ophthalmic training, potentially introducing variability in screening quality, referral decisions, and follow-up practices [9]. These challenges highlight the need for accessible, standardized, and scalable approaches capable of strengthening existing school eye health programs while improving referral pathways and continuity of care.

School-based vision screening has traditionally relied on conventional VA charts administered by trained personnel, an approach that can be resource-intensive and difficult to implement consistently at scale [10]. In recent years, smartphone- and tablet-based VA applications, including Peek Acuity and WHOeyes, have demonstrated good agreement with conventional VA assessment and offer practical alternatives for school- and community-based screening, particularly in resource-limited settings [11]. These digital tools have improved the accessibility and standardization of VA assessment while reducing dependence on specialized equipment and trained eye care personnel [12]. However, successful implementation of school vision screening requires substantially more than accurate VA measurement alone. Large-scale programs depend on standardized referral pathways, secure data management, longitudinal follow-up, program monitoring, and integration with existing health and education systems. Although the technical performance of digital VA applications has been evaluated extensively, evidence regarding their implementation within routine school health systems remains limited, particularly in low- and middle-income countries. Implementation outcomes such as workflow integration, usability by non-eye-care personnel, and operational feasibility are therefore important considerations alongside screening performance.

To address these implementation gaps, we developed CERMATA, an Indonesian web-based digital school vision screening platform that incorporates the validated WHOeyes mobile application within a broader referral and program management platform. In addition to automated VA screening, the platform incorporates participant registration, referral management, centralized reporting, longitudinal follow-up, and psychosocial screening using the Strengths and Difficulties Questionnaire (SDQ-25). CERMATA was specifically designed to support school-based screening conducted by trained non-eye-care personnel while maintaining standardized testing procedures suitable for large-scale implementation within the Indonesian education and healthcare systems.

The present study evaluated the pilot implementation and preliminary screening performance of the CERMATA platform under routine school conditions rather than re-validating the underlying WHOeyes VA algorithm. Accordingly, this study focused on older primary school children, among whom myopia and unmet refractive needs become increasingly prevalent. Specifically, this study evaluated (1) the preliminary operational feasibility of implementing the platform in Indonesian primary schools, (2) the agreement between CERMATA-generated referral decisions and standardized Snellen VA screening, and (3) the preliminary screening performance of the platform for identifying children with referral-level reduced VA requiring further ophthalmic assessment. Consistent with implementation science methodology, this pilot phase was intended to evaluate preliminary operational feasibility and generate initial estimates of screening performance rather than provide definitive validation estimates. By integrating VA screening with referral management and program monitoring within a single digital platform, CERMATA may facilitate more standardized implementation and continuity of care across schools and healthcare settings. The findings are intended to inform refinement of the platform and the design of subsequent multicenter implementation and validation studies across more diverse populations and healthcare settings.

## Methods

### Study design

This cross-sectional pilot implementation study evaluated the preliminary operational feasibility and screening performance of CERMATA, a web-based digital school vision screening platform, during implementation in Indonesian primary schools. The study was conducted between May and October 2025 in two primary schools participating in the initial implementation phase of the CERMATA program in Jakarta, Indonesia. The study was designed primarily to assess the feasibility of integrating the platform into routine school-based screening workflows and secondarily to generate preliminary estimates of screening performance to inform the design of subsequent multicenter validation studies. Reporting of screening performance measures followed the Standards for Reporting Diagnostic Accuracy Studies (STARD) 2015 recommendations where applicable to screening studies.

### Participants and sample size

Participants were selected from the CERMATA pilot implementation cohort, which included 849 students aged 9–12 years enrolled in grades 4–6 at 15 primary schools in Jakarta, Indonesia. For the present study, two of the 15 participating schools were randomly selected from the pilot implementation study. Children were eligible if written informed consent was obtained from parents or legal guardians and verbal assent was obtained from the child. Children were excluded only if informed consent was withdrawn, severe cognitive or communication impairment prevented reliable VA assessment, significant ocular pathology affecting VA independently of refractive status was present (including visually significant cataract, corneal opacity, or severe ptosis), or outcome data were incomplete. Participants who did not complete one or both screening procedures were retained in the participant flow diagram in accordance with STARD recommendations [13] and excluded from paired screening performance analyses.

Sample size for the definitive validation study was estimated at 362 participants based on anticipated sensitivity, specificity, disease prevalence, a 95% confidence level, and a precision of 10%. Consistent with recommendations for pilot implementation studies, approximately 10–20% of the estimated sample size for the definitive validation study was considered adequate for evaluating operational feasibility and generating preliminary estimates of screening performance [14]. A minimum sample size of 37 participants was therefore considered sufficient for the present pilot phase.

### Development of the CERMATA platform

Development of the CERMATA platform was informed by a preliminary mixed-methods study conducted in 15 primary schools in Jakarta involving 1,254 students and 97 stakeholders, including teachers, parents, and primary healthcare workers. Findings from this phase informed subsequent refinement through multidisciplinary expert consultation and field testing. CERMATA incorporates the WHOeyes mobile application within a broader digital platform that includes referral management and psychosocial screening using the Strengths and Difficulties Questionnaire (SDQ-25).

CERMATA is a web-based VA screening system adapted from the WHOeyes framework and employs tumbling-E optotypes displayed through standard web browsers on Android and iOS devices. Optotype dimensions were automatically scaled according to screen size and resolution using the WHOeyes calibration protocol to maintain a constant angular subtense across devices. Before each screening session, the built-in calibration procedure was completed to ensure accurate optotype scaling. Standard mobile internet connectivity was required to access the web-based platform and synchronize screening data with the central database during testing.

Prior to implementation, 128 facilitators consisting of teachers and parents completed standardized training on platform operation, screening procedures, and referral workflows. All personnel involved in data collection completed standardized training covering participant positioning, monocular VA assessment procedures, occlusion techniques, device calibration, environmental preparation, referral procedures, and electronic data entry requirements. The refined platform was subsequently piloted in 849 students before initiation of the present study.

### Visual acuity assessment

All participants underwent VA assessment using the CERMATA platform followed immediately by conventional Snellen chart testing on the same day. CERMATA screening was consistently performed first to maintain a uniform workflow during school implementation, and the order of testing was not randomized. Snellen examiners were masked to CERMATA screening results, while facilitators conducting CERMATA screening remained unaware of Snellen findings during testing.

VA was assessed monocularly with the fellow eye fully occluded. The right eye was tested first, followed by the left eye, and habitual spectacle correction was worn when available. Testing was performed at a viewing distance of 2 m measured using a calibrated measuring tape. Assessments were conducted indoors under routine classroom lighting conditions while avoiding direct glare and screen reflections. Ambient illumination was not quantitatively standardized because the objective of the pilot phase was to evaluate performance under routine implementation conditions.

Facilitators provided standardized instructions, adjusted screen brightness to near-maximum intensity, and devices were positioned perpendicular to the participant’s eye level. Referral recommendations were generated automatically by the embedded screening algorithm without examiner interpretation or modification. Participants with monocular VA worse than 6/9 (0.18 logMAR) in either eye were classified as screening positive and referred for comprehensive ophthalmic assessment [15].

The reference assessment consisted of conventional Snellen VA testing performed independently using standardized Snellen charts at 6 meters under the same testing conditions. The test was conducted by an ophthalmologist and an optometrist experienced in standardized VA measurement.

### Statistical analysis

All analyses were performed at the participant level to reflect the referral process used in school-based vision screening and to account for inter-eye correlation. For both the CERMATA platform (index test) and the Snellen VA assessment (reference standard), the eye with the poorer presenting VA was selected for analysis. Participants meeting the referral criterion in either eye were classified as screening positive, consistent with routine school vision screening practice.

Screening performance was evaluated by calculating sensitivity, specificity, positive predictive value, negative predictive value, positive likelihood ratio (LR+) and negative likelihood ratio (LR−), overall accuracy, and the Youden index, each with corresponding 95% confidence intervals. Agreement between the CERMATA platform and the reference standard was assessed using Cohen’s kappa coefficient. Receiver operating characteristic (ROC) curve analysis was performed using continuous presenting VA values obtained from the CERMATA platform to estimate the area under the curve (AUC).

All statistical analyses were performed using IBM SPSS Statistics for Windows, Version 27. All tests were two-sided, and a p value <0.05 was considered statistically significant.

### Ethics

The study was approved by the Institutional Ethics Committee of Universitas Katolik Indonesia Atma Jaya (Approval No. 007E/III/PPPE.PM.10.05/7/2024). Written informed consent was obtained from the parents or legal guardians of all participants prior to study enrollment, and verbal assent was obtained from participating children before study procedures. All procedures were conducted in accordance with the principles of the Declaration of Helsinki.

## Results

For the present screening performance evaluation, two schools were randomly selected from the CERMATA pilot implementation cohort, comprising 206 children. Of these, 33 children did not attend on the assessment day and therefore had no examination data, leaving 173 children who completed the CERMATA screening. Among these, 72 children underwent both CERMATA screening and reference Snellen visual acuity assessment and were included in the paired screening performance analysis (Fig 1). The 72 participants included in the paired screening performance analysis comprised 52 females (72.2%) and 20 males (27.8%), with ages ranging from 9 to 12 years.

**Fig 1:**
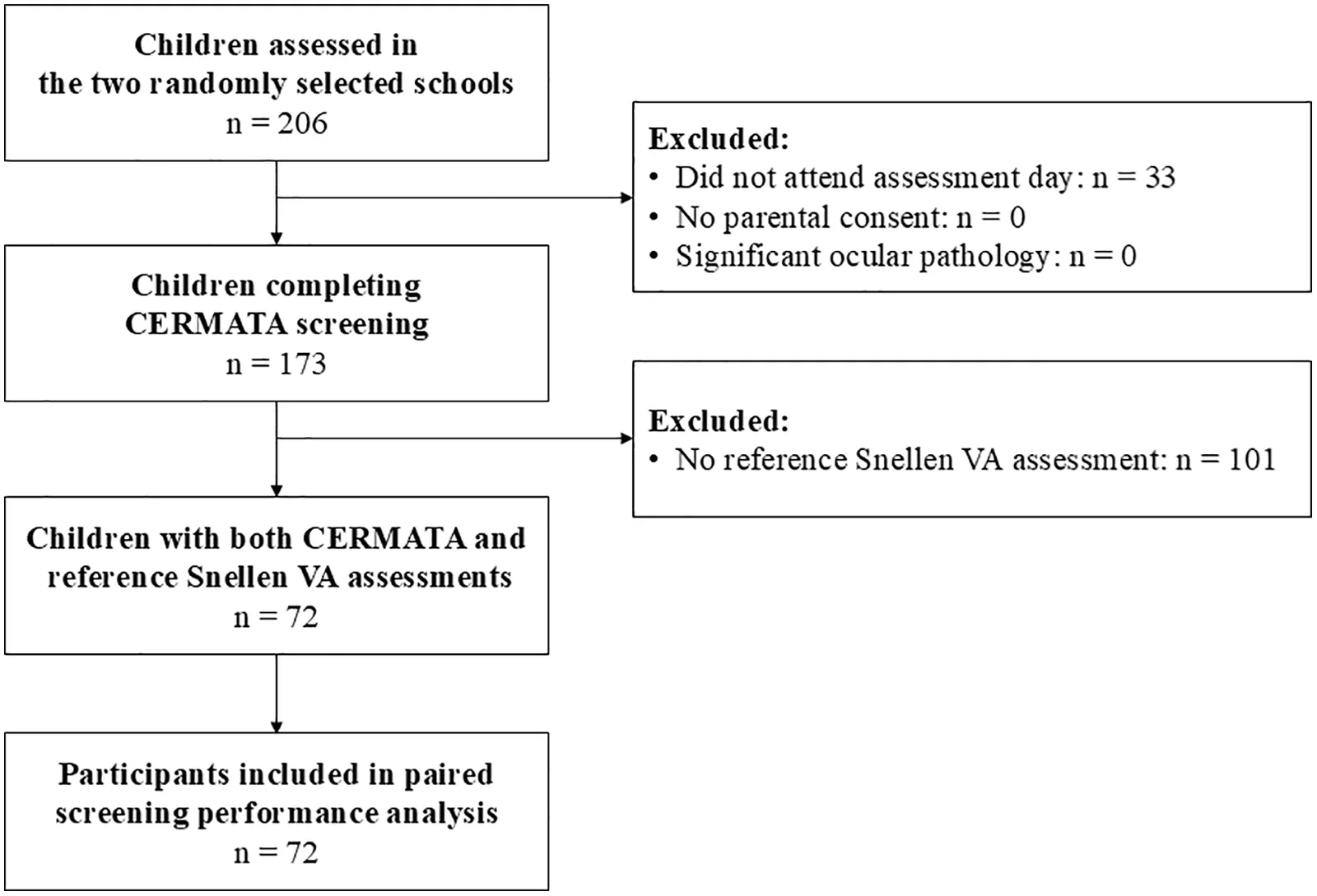
STARD flow diagram of participant recruitment and screening performance analysis. The diagram illustrates participant selection from the CERMATA pilot implementation cohort, eligibility assessment, completion of the index test (CERMATA) and reference standard (Snellen VA assessment), participant exclusions, and inclusion in the participant-level screening performance analysis using the worse eye.

Presenting VA was assessed using the Snellen chart and the CERMATA platform. Participants were classified as having referral-level reduced VA if presenting VA was worse than 6/9, whereas VA 6/9 or better in both eyes was classified as normal or non-referable. Based on the reference standard, 58 participants (80.6%) met referral criteria, whereas 14 participants (19.4%) were classified as non-referable. According to the CERMATA platform, 58 participants (80.6%) were classified as having referral-level reduced VA, while 14 participants (19.4%) were classified as non-referable.

As shown in Table 1, the CERMATA platform correctly identified 56 of 58 participants with referral-level reduced VA and correctly classified 10 of 14 participants without referral-level reduced VA. The CERMATA platform demonstrated a sensitivity of 96.6% and a specificity of 71.4% for detecting reduced presenting VA compared with the Snellen reference standard. The positive predictive value (PPV) was 93.3%, while the negative predictive value was 83.3%, resulting in an overall accuracy of 91.7%. The positive likelihood ratio (LR+) was 3.38, indicating that participants with reduced presenting VA were more than three times as likely to have a positive CERMATA screening result than those without reduced presenting VA. The negative likelihood ratio (LR–) was 0.05, indicating that a negative screening result substantially reduced the probability of reduced presenting VA. The Youden index was 0.680, indicating good overall discriminatory performance of the screening test.

**Table 1:** Two-by-two contingency table comparing CERMATA screening results with the Snellen reference standard.

	Snellen Positive	Snellen Negative	Total
**CERMATA Positive**	56 (True Positive)	4 (False Positive)	60
**CERMATA Negative**	2 (False Negative)	10 (True Negative)	12
	58	14	72

Agreement between CERMATA referral classifications and standardized Snellen VA assessment was substantial, with a Cohen’s kappa coefficient of 0.719 (p < 0.001).

ROC analysis was subsequently conducted using the continuous CERMATA score from the worse eye against the binary classification of reduced presenting VA derived from the Snellen reference standard. The ROC curve demonstrated strong discriminative ability, with an AUC of 0.914 (95% CI 0.83–1.00; p < 0.001), indicating excellent overall screening performance (Fig 2). An AUC above 0.90 suggests that CERMATA has a high probability of correctly distinguishing children with reduced VA from those with normal vision.

**Fig 2:**
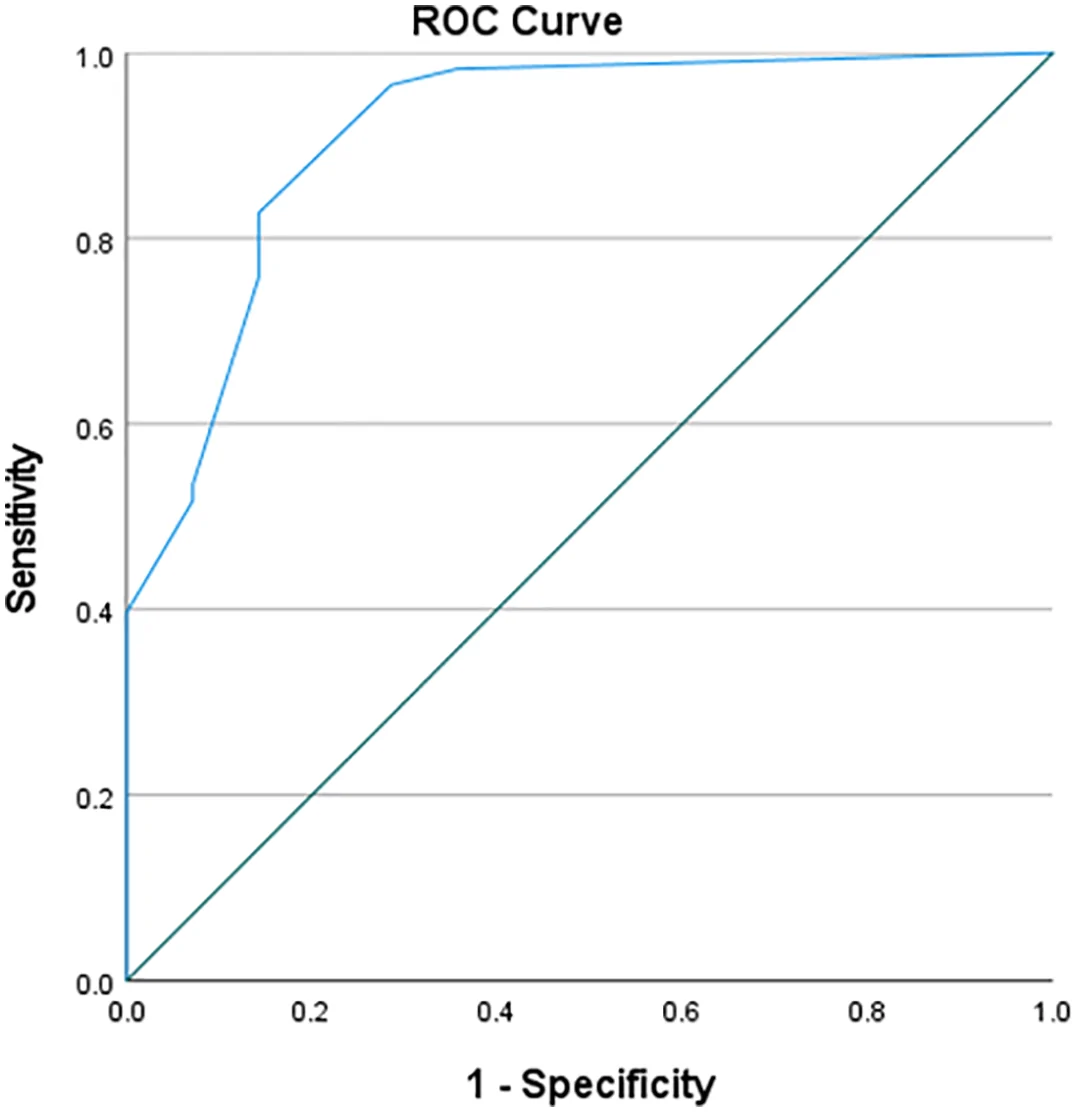
Receiver operating characteristic (ROC) curve of the CERMATA platform for detecting referral-level reduced VA. The ROC analysis was performed using the continuous CERMATA score from the worse eye against the binary classification of referral-level reduced VA based on the Snellen reference standard. The area under the ROC curve (AUC) was 0.914 (95% CI 0.83–1.00; *p* < 0.001).

All 72 participants included in the paired screening performance analysis successfully completed both the CERMATA assessment and the reference Snellen VA assessment, resulting in a completion rate of 100% with no missing outcome data, supporting the feasibility of implementing the screening workflow under field conditions. Following standardized training, non-eye-care personnel were able to perform the screening procedures and operate the platform according to the study protocol.

## Discussion

The need for such an integrated platform may be particularly relevant in Indonesia, where geographical dispersion and inequalities in access to eye care services continue to affect service delivery in many regions [16,17]. The World Report on Vision highlighted substantial disparities in the availability and accessibility of eye care services globally, particularly in low- and middle-income countries, and emphasized the need for integrated people-centered eye care systems to improve service delivery and continuity of care [18]. In school-based screening programs, limited personnel and paper-based documentation systems may further complicate referral monitoring and long-term follow-up [19,20]. Previous studies have emphasized that the success of school vision screening depends not only on the accuracy of VA assessment but also on efficient referral systems that ensure appropriate management after screening [10,21]. Future implementation studies are warranted to evaluate its long-term effectiveness, acceptability, cost-effectiveness, and scalability when integrated into routine school health services across diverse regions of Indonesia.

This pilot study included primary school children aged 9–12 years (grades 4–6), although vision screening is generally recommended for younger children aged 4–6 years to facilitate the early detection of visual disorders before school entry. The older age group was intentionally selected because children in this age range are generally more independent and cooperative during vision assessment and can complete screening procedures without parental or caregiver assistance [22]. Furthermore, the choice of this age group is supported by the increasing prevalence of refractive errors, particularly myopia, during late childhood [23,24]. Myopia commonly develops and progresses during the primary school years, making children aged 9–12 years an important target population for school-based vision screening [24]. Therefore, evaluating CERMATA in this age group provides a practical assessment of its performance in a population with a relatively high prevalence of visual impairment while reflecting the real-world context in which school-based vision screening programs are frequently implemented.

This pilot study demonstrated favorable screening performance of CERMATA for identifying children with referral-level reduced VA while supporting standardized school-based vision screening through an integrated digital platform. CERMATA demonstrated high sensitivity (96.6%) and specificity (71.4%), substantial agreement with standardized Snellen VA assessment (κ = 0.719), and excellent discrimination on ROC analysis (AUC = 0.914). The relatively high sensitivity is particularly desirable in school-based screening because the primary objective is to minimize missed children who require further ophthalmic assessment [25]. Failure to identify children with reduced VA may delay intervention and adversely affect educational performance and quality of life [26,27]. Although high sensitivity may result in false-positive referrals, this trade-off is generally acceptable in screening programs when the consequences of missed cases are more substantial than those of unnecessary referrals [28]. In Indonesia, the national health insurance system, administered by BPJS Kesehatan, requires patients to undergo initial evaluation at a primary healthcare center (Puskesmas) before referral to an ophthalmologist or optometrist when indicated, providing an additional layer of triage [29,30]. This mechanism may help limit the burden of false-positive referrals while preserving the benefits of early identification and timely access to eye care.

This study has several limitations. First, because the objective was the identification of referral-level reduced VA rather than the diagnosis of refractive error, standardized Snellen VA assessment was used as the reference standard, and comprehensive ophthalmic examination, including cycloplegic refraction, was not performed in all participants. Furthermore, because both CERMATA and the reference standard assess presenting VA using similar psychophysical constructs, incorporation or construct overlap bias may have contributed to the observed agreement estimates. Second, inter-rater agreement was not formally assessed, although automated VA calculations and referral recommendations are expected to reduce operator-dependent variability. Third, the relatively small sample size and the high prevalence of referral-level reduced VA within the implementation cohort may limit the generalizability of the findings and may have influenced positive predictive value estimates. Fourth, the study was conducted in only two primary schools in Jakarta, which may further limit the generalizability of the findings to other geographic, socioeconomic, and educational settings in Indonesia. Finally, although CERMATA functioned adequately across different devices, formal comparisons between device types were not performed. Future multicenter studies involving larger and more diverse populations are warranted to confirm the reproducibility, generalizability, and implementation feasibility of CERMATA in routine school-based vision screening programs.

## Conclusion

CERMATA demonstrated promising screening and substantial agreement with conventional VA assessment in this pilot sample. Its digital referral-based design supports feasibility for scalable school-based screening and may contribute to improved identification of children with referral-level reduced VA. Multicenter implementation studies involving larger and more diverse populations are required to confirm its performance, scalability, and integration into routine eye-care pathways before national-scale implementation.

## Supporting information

S1 File STARD 2015 checklist. (DOCX)

S1 Data Anonymized dataset. (XLS)
