# Supplementary material for: Development and pilot implementation of CERMATA: A digital referral-based vision screening platform for Indonesian primary school children
Source: PLoS One. 2026 Sep 30;21(9):e0359572. doi: 10.1371/journal.pone.0359572 (PMC13626466; doi:10.1371/journal.pone.0359572)
Supplement: Supplementary file 1 [file pone.0359572.s001.docx]

**STARD 2015 Checklist**

| **Section & Topic** | **No.** | **STARD 2015 Item** | **Reported on page(s) / Comments** |
| --- | --- | --- | --- |
| **TITLE OR ABSTRACT** | **1** | Identification as a study of diagnostic accuracy using at least one measure of accuracy (e.g., sensitivity, specificity, predictive values, AUC) | **Pages 1–2** – The Abstract reports the study as a pilot implementation study and presents screening performance measures, including sensitivity, specificity, predictive values, and AUC. |
| **ABSTRACT** | **2** | Structured summary of study design, methods, results, and conclusions | **Page 2** |
| **INTRODUCTION** | **3** | Scientific and clinical background, including intended use and clinical role of the index test | **Pages 2–4** |
|  | **4** | Study objectives and hypotheses | **Page 4** |
| **METHODS – Study design** | **5** | Whether data collection was planned before the index test and reference standard were performed | **Page 5 –** Cross-sectional pilot implementation study. |
| **Participants** | **6** | Eligibility criteria | **Page 5** |
|  | **7** | Basis on which potentially eligible participants were identified | **Page 5** – Selected from the CERMATA pilot implementation cohort. |
|  | **8** | Where and when potentially eligible participants were identified | **Page 5 –** The study was conducted between May and October 2025 in two primary schools in Jakarta, Indonesia. |
|  | **9** | Consecutive, random, or convenience series | **Page 5** – Two schools randomly selected from the implementation cohort. |
| **Test methods** | **10a** | Index test, in sufficient detail to allow replication | **Pages 5–7** |
|  | **10b** | Reference standard, in sufficient detail to allow replication | **Page 7** |
|  | **11** | Rationale for choosing the reference standard | **Pages 7 & 10** – Standardized Snellen visual acuity assessment was selected as the reference standard because it represents the conventional method for school-based visual acuity screening and aligns with the study objective of identifying referral-level reduced visual acuity rather than establishing an ophthalmic diagnosis. |
|  | **12a** | Definition and rationale for index test positivity cut-off | **Page 7** – Referral threshold >6/9 (0.18 logMAR). |
|  | **12b** | Definition and rationale for reference standard positivity cut-off | **Pages 7** – Same referral threshold applied to the reference assessment. |
|  | **13a** | Whether clinical information/reference results were available to performers/readers of the index test | **Page 6** – Facilitators were blinded to Snellen findings. |
|  | **13b** | Whether clinical information/index test results were available to assessors of the reference standard | **Page 6** – Snellen examiners were masked to CERMATA results. |
| **Analysis** | **14** | Methods for estimating or comparing diagnostic accuracy | **Page 7** |
|  | **15** | How indeterminate index or reference results were handled | **Not applicable.** The predefined referral threshold classified all participants as either screening positive or screening negative; therefore, no indeterminate index or reference standard results occurred. |
|  | **16** | How missing data were handled | **Page 9 –** Among the 72 participants included in the paired screening performance analysis, all completed both the index test and reference standard, with no missing outcome data. |
|  | **17** | Analyses of variability in diagnostic accuracy | **Not applicable.** No prespecified or exploratory analyses of variability in screening performance were performed. |
|  | **18** | Intended sample size and how it was determined | **Page 5** |
| **RESULTS – Participants** | **19** | Flow of participants using a diagram | **Pages 8 & 10 (Fig. 1)** |
|  | **20** | Baseline demographic and clinical characteristics | **Page 8** |
|  | **21a** | Distribution of severity of disease in those with the target condition | **Not applicable.** The study evaluated referral-level reduced visual acuity as a screening outcome rather than disease severity, and no severity classification of the target condition was performed. |
|  | **21b** | Distribution of alternative diagnoses in those without the target condition | **Not applicable.** Comprehensive ophthalmic examination was not performed; alternative diagnoses were not established. |
|  | **22** | Time interval and any clinical interventions between index test and reference standard | **Pages 6** – Index and reference assessments were performed on the same day; no clinical interventions occurred between tests. |
| **RESULTS – Test results** | **23** | Cross-tabulation of index test by reference standard | **Page 11 (Table 1)** |
|  | **24** | Estimates of diagnostic accuracy and precision | **Page 8**– Sensitivity, specificity, positive and negative predictive values, likelihood ratios, Cohen's κ, AUC, and 95% confidence intervals reported. |
|  | **25** | Any adverse events from performing the index test or reference standard | **Not applicable.** No adverse events occurred during non-invasive visual acuity assessment. |
| **DISCUSSION** | **26** | Study limitations, including sources of potential bias, statistical uncertainty, and generalisability | **Pages 9-10** |
|  | **27** | Implications for practice, including intended use and clinical role of the index test | **Pages 9–11** |
| **OTHER INFORMATION** | **28** | Registration number and name of registry | **Not applicable.** This was an observational cross-sectional pilot implementation study and was not registered in a clinical trial registry. |
|  | **29** | Where the full study protocol can be accessed | **Not applicable.** A publicly accessible study protocol is not available. |
|  | **30** | Sources of funding and other support; role of funders | **Reported in the Financial Disclosure section of the PLOS ONE submission system and reiterated in the Cover Letter, in accordance with PLOS ONE funding disclosure policy.** The development and implementation of the CERMATA platform was funded by the Good Neighborhood Program from Ford Foundation Indonesia through the Indonesia International Education Foundation (IIEF). The funders had no role in study design, data collection and analysis, decision to publish, or preparation of the manuscript, in accordance with PLOS ONE reporting requirements. |
